# Interpersonal Harmony and Conflict for Chinese People: A Yin–Yang Perspective

**DOI:** 10.3389/fpsyg.2016.00847

**Published:** 2016-06-08

**Authors:** Li-Li Huang

**Affiliations:** Department of Psychology, National Taiwan University, TaipeiTaiwan

**Keywords:** Chinese indigenous psychology, conflict, emotionalizing, harmony, Yin–Yang perspective

## Abstract

This article provides an overview on a series of original studies conducted by the author. The aim here is to present the ideas that the author reconstructed, based on the dialectics of harmonization, regarding harmony and conflict embodied in traditional Chinese thought, and to describe how a formal psychological theory/model on interpersonal harmony and conflict was developed based on the Yin–Yang perspective. The paper also details how essential theories on interpersonal harmony and conflict were constructed under this formal model by conducting a qualitative study involving in-depth interviews with 30 adults. Psychological research in Western society has, intriguingly, long been focused more on interpersonal conflict than on interpersonal harmony. By contrast, the author’s work started from the viewpoint of a materialist conception of history and dialectics of harmonization in order to reinterpret traditional Chinese thought. Next, a “dynamic model of interpersonal harmony and conflict” was developed, as a formal psychological theory, based on the real-virtual notions in the Yin–Yang perspective. Under this model, interpersonal harmony and conflict can be classified into genuine versus superficial harmony and authentic versus virtual focus conflict, and implicit/hidden conflict is regarded as superficial harmony. Subsequently, the author conducted a series of quantitative studies on interpersonal harmony and conflict within parent–child, supervisor–subordinate, and friend–friend relationships in order to verify the construct validity and the predictive validity of the dynamic model of interpersonal harmony and conflict. The claim presented herein is that Chinese traditional thought and the psychological theory/model based on the Yin–Yang perspective can be combined. Accordingly, by combining qualitative and quantitative empirical research, the relative substantial theory can be developed and the concepts can be validated. Thus, this work represents the realization of a series of modern Chinese indigenous psychological research studies rooted in traditional cultural thought and the Yin–Yang perspective. The work also mirrors the current conflict-management research that has incorporated the Chinese notion of harmony and adopted the Yin–Yang perspective on culture.

## Introduction

This article provides an overview on a series of research studies that the author conducted on interpersonal harmony and conflict. The primary objective here is to present a method to develop a formal psychological theory/model regarding interpersonal harmony and conflict founded on one specific aspect of Eastern philosophy, the Yin–Yang perspective. Moreover, the work discussed in this paper shows that by combining qualitative and quantitative empirical research, the substantial theory can be developed and its underlying concepts can be validated. According to the definition and typology of Li (2012), this theory/model and the related series of research studies could be attributed to the “Eastern *emic-*as-*emic*” type of indigenous research, in which the aim is to build novel Eastern theories that complement/supplement or supersede Western theories.

Conflict has invariably been a common topic of interest in the social sciences and in psychology because conflict is inevitable in human society. Numerous psychological studies have examined conflict: A search of the APA PsycINFO database in 2015 retrieved 73,167 papers on conflict and conflict resolution, and from 2007 onward, 2,000–2,500 research papers have been published annually on this subject, which shows that conflict is a major topic of debate in psychology. By contrast, only 1,422 papers on harmony have been published to date, and since 2012, less than 100 papers related to harmony have been published annually. It is intriguing why the publications about harmony are 50 times fewer than the publications about conflict.

The Chinese philosopher Cheng (1977) indicated that, from a metaphysical perspective, the prevailing dialectic of conflict in the contemporary West was inherited from Hegel and Marx, which contrasted the focus of Confucianism and Taoism on the dialectic of harmonization. These dialectical views differ in their ontological assumptions (realism) and in the logic and purpose of their reflections. A marked distinction also exists between their cultural backgrounds and problem-solving methods (conflict resolution). The following section briefly introduces conflict and its development within a Western context.

### Western View of Conflict and Its Evolution

The Western view of conflict has evolved through its interaction with various perspectives. Since the time of Plato and Aristotle, maintaining order has been considered beneficial for a society, and conflict-induced chaos has been regarded to pose a threat to state and society (Rahim, 1986). In the 19th century, Darwin used the phrase “survival of the fittest,” with the underlying suggestion being that species survive and grow by coping with the challenges presented by the surrounding environment. This concept, when applied to human society, indicates that conflicts between humans and the environment are opportunities for humanity to evolve.

In the middle of 20th century, the American sociologist T. Parsons (1949) proposed the theory of structural functionalism, which suggests that the core features of society are stability, integration, and functionality. Here, conflict is considered a “functional disorder” that includes destruction, division, and dysfunction, and is regarded as a social disease. By contrast, the sociologist Simmel (1908–1955>) held that moderate conflict, like order and cooperation, commonly performs a positive function in, and can facilitate the formation and sustainability of societies or groups. This perspective coincided with the view of **conflict dialectics**: The existence of two opposites, thesis and antithesis, is an objective fact, and the conflict between these opposites leads to an elevated level of synthesis after coordination, and the world can continuously develop toward an “ideal” world by following the same path, in a spiral manner. Since this proposal, the perspective of conflict as a constructive function has dominated the field of social science. However, this perspective mainly focuses on society (organization) and groups, and further clarification of this view is required if it is to be applied to interpersonal relationships or scenarios that entail interpersonal conflict.

Western psychological studies have primarily focused on individual mind and behavior and have thus considered conflict at an individual level. For example, Freud’s psychoanalytic theory is a psychology of conflict (Luborsky et al., 2011): Freud noted that people are inevitably caught between two opposing forces that cannot be changed, as in the case of the conflict between the id and the ego or between the id and the super-ego. A person’s mental health condition depends on adjusting these two opposing forces while concurrently maintaining their dynamic balance, and a loss of this balance might cause conflict and, subsequently, lead to neurosis (Fenichel, 1945). Interpersonal conflict results from people transferring their inherent conflict to interpersonal relationships. In summary, conflict is an internal state that causes tension or anxiety when a person is unable to clearly decide between two opposing goals.

The definition of dyadic conflict (including interpersonal, intergroup, and international conflicts) appears contradictory; however, the common accepted feature of conflict is that it is *interactive* (Rahim, 1986). Conflict, by definition, includes opposition, scarcity, and blockage; thus, conflict must involve at least two opposing parties whose interests, goals, and benefits are incompatible. Resources such as money, status, and power are limited, and both parties in a conflict are responsive with regard to emotion, cognition, and behavior.

The prisoner’s dilemma game (PDG) is the most widely used model for dyadic conflict in social psychology studies in a laboratory setting. In follow-up studies conducted since the PDG was developed, researchers converted competitive reactions to the PDG into “pursuing personal goals” and thereby converted cooperative reactions into “considering interpersonal relationships.” Subsequently, five conflict resolutions were proposed, competition, accommodation, avoidance, integration, and compromise (Hall, 1969; Filley, 1975; Thomas, 1976), as were two conflict results, lose–lose and win–win. The five categories of conflict resolution were derived from the social exchange theory, which assumes that human behavior is rational and its aim is the pursuit of maximal profit. Thus, the model of conflict resolution could be discussed suitably within the context of “realistic conflict.” However, the five categories emphasize rationality while neglecting sentiment, and they have been used to justify traditional psychology when conducting quantitative empirical research and analyzing cause and effect. It is a drawback that the model of conflict resolution was thus changed from a dynamic model into a static model.

However, Braithwaite (1997) conducted a study in Australia that included 197 university students (aged 17–64 years), and the results showed that “pursuit of harmony” was a crucial value that connected people, interpersonal relationships, and social groups. Moreover, by studying “wisdom” as it is commonly recognized by adults, Jason et al. (2001) determined that “harmony” is one of the five most critical relationship factors. Furthermore, Kwan et al. (1997) indicated that both “maintaining harmonious relationships” and “self-esteem” are intermediate variables that affect life satisfaction. These studies indicate that research perspectives have gradually eluded the restrictions of the Western view of conflict.

In other studies, Leung and Brew (2009) performed a cultural analysis on harmony and conflict, and Leung et al. (2002) developed a dualistic model of harmony; in this model, harmony included harmony enhancement and disintegration avoidance, and the model was used for investigating the differences between Chinese and Australian people in terms of the two harmony facets (Leung et al., 2011). Furthermore, Hwang (1997–1998) argued that pursuing goals and maintaining harmony are mutually exclusive in Chinese conflict resolution. Although these models and research studies considered conflict and harmony concurrently, they mostly emphasized conflict resolution or management without adopting the Yin–Yang perspective.

### Value of Harmony in Chinese Culture and Its Influence

Chinese people hold a deep-rooted desire to pursue “harmony” when cultivating one’s self, handling interpersonal matters, and confronting the universe and nature. The Chinese anthropologist Lee (1992) indicated that Chinese culture is based on pursuing harmony and balance and maintaining them within three subsystems: nature (heaven), organisms (humans), and interpersonal relationships (society). In an analysis of the data on social change in Taiwan, Huang and Chu (2013) indicated that since 1985, “harmony” has been considered the most critical underlying value. Shek and Daniel (2001) studied 400 Chinese parents and teenagers, and reported that all study participants agreed that harmony and a lack of conflict were the most crucial elements of a “happy” family. Chinese people have been widely suggested to be typically peaceable, submissive, and friendly, and to dislike resistance, defiance, opposition, competition, and fighting (Wen, 1972; Yang, 1972; Sun, 1983; Liang, 1989). Chinese people commonly link “conflict” with “turmoil” and thus tend to dislike conflict and even avoid it out of fear. Pye (1992, 1982) indicated that the Chinese political culture and bureaucracy strongly emphasize maintaining order and avoiding conflict.

The widely recognized Chinese philosopher Feng (1991) indicated, in *History of Chinese Philosophy*, that dialectics can end not only in conflict but also in harmony because the universe mainly consists of *tai he* (unification). This mirrors the theory of Cheng (1977) that Chinese culture, Confucianism and Taoism in particular, is the metaphysic of harmony and conflict and the dialectics of harmonization based on the Yin–Yang perspective.

Yin–Yang is both an indigenous and traditional notion and a type of Chinese philosophy, and it could be regarded as a symbol (Fang, 2012), a dialectical logic system (Li X., 2014), a cognitive frame (Li, 2012), or an epistemology. However, in modern social sciences, Yin–Yang is typically presented as a perspective, particularly a perspective on Eastern culture (Fang, 2012). As a symbol, Yin and Yang might represent any pair of dichotomous categories or elements, such as male and female, good and bad, top and bottom, right and left, and black and white, but the two contrary elements can be mutually transformed. However, Li P.P. (2014) noted that the application of the Yin–Yang frame is context-specific rather than context-free.

Harmony and conflict could correspond to Yin and Yang, and most concepts in Chinese philosophy can be conceived and evaluated in a framework of harmony and conflict as two modes of thinking, two orientations, or two aspects of a changing reality (Cheng, 1977). Therefore, Cheng (1977) also suggested a metaphysical view of harmony and conflict, and, consequently, proposed that “the dialectics of harmonization” is suitable for denoting Chinese thought, such as Confucianism and Taoism, and is logically distinct from both Hegel’s dialectics of conflict and Madhyamika dialectics. The Merriam-Webster dictionary defines metaphysics as a division of philosophy that is concerned with the fundamental nature of reality and being and which includes ontology, cosmology, and often epistemology. According to the notion proposed by Cheng (1977), a metaphysic will determine the manner in which objects in the world are viewed and will also present a method of solving problems and conducting inquiry.

The preceding discussion suggests that the Cheng (1977) view of “dialectics of harmonization” considers “harmony” and “conflict” to be mutually defined categories that also involve mutual relationships. Moreover, the entire universe, human society, and individual life are all considered to incline toward harmony and unification: Conflict is an unnatural disorder and an imbalance. The diversity and duality of entities are the causes of conflict and opposition. However, complementary and alternating elements exist within conflict and opposition: two opposing parties are equal overall and might reach harmony through regulation. In this regard, harmony is not a static structure but a dynamic process. Harmony is the ultimate ideal and goal, and in the pursuit of harmony, the resolution of potential conflicts can be found within the mechanism of harmonization.

In summary, “harmony” is the core concept and value of Chinese culture. Furthermore, from the perspective of Yin–Yang dialectics, the concept of harmony is correlated with conflict. Therefore, to comprehensively understand the interpersonal conflicts of Chinese people, we must begin by examining interpersonal harmony. The following section describes how harmony and conflict among Chinese people were clarified through an exploration of the history of China and its cultural context, and how a dynamic model of interpersonal harmony and conflict was constructed based on the Yin–Yang perspective.

## Huang’s “Dynamic Model of Interpersonal Harmony and Conflict”

### Concepts and Implications of Harmony and Conflict among Chinese People

Disregarding the traditional approach used for categorizing culture and schools of thought (Confucianism, Legalism, and Taoism), Huang (1999) delineated three traditional Chinese (cultural) ideologies from the perspective of substructures affecting superstructures (i.e., by using a historical materialism approach). In Huang’s view, the structure of traditional Chinese society primarily entailed an agricultural society bound by kinship under an extensive but unified political and religious system. The superstructures that corresponded with these three substructures were the integration of heaven and humanity (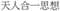), the theory of rituals (*Li*-thoughts, 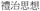), and a state-ideological Confucianism (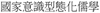). The “integration of heaven and humanity” defines the relationship between humanity and nature; Chinese people claim that, at the universal level, the relationship of humanity and nature is one of dynamic harmony, and that by following the law of nature, humans can pursue inner freedom and peace. The “theory of rituals,” which entails interpersonal ethics, describes how people must discipline themselves and express their emotions within various relationships in a manner that achieves interpersonal harmony. The standard used for these relationships is the Five Cardinal Relationships (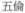): ruler–subject, father–son, brother–brother, husband–wife, and friend–friend relationships. The “state-ideological Confucianism” is applied distinctly at various levels of groups, organizations, and societies; when seeking to achieve a collective goal, complying with normative roles benefits the most number of people in a group.

The aforementioned three perspectives of harmony involve the ideals of individual personality, interpersonal ethics, and the interests and effectiveness of groups and organizations. These perspectives imply that the results of adjustments and efficacy can transform harmony from a dynamic and carefree state into a static and formal one. Thus, the Chinese people’s concept of harmony manifests itself dissimilarly at distinct levels; this implies that the states of harmony differ at various levels and in distinct interpersonal relationships, and thereby demonstrates the existence of diverse methods that can be used for reaching harmony. Corresponding with these three concepts of harmony are the Chinese people’s three concepts of conflict: in a conflict, a person drops to a morally inferior position, maintains a losing stance emotionally and rationally, and pays a high price in personal, societal, and survival aspects.

In summary, for Chinese people, harmony is the most crucial core concept, and their concepts of harmony are correspondingly complex and diverse. Therefore, considerably more knowledge can be gained by examining ethnic Chinese people’s harmonization mechanisms than from exploring their methods of conflict resolution.

### Building a Formal Psychological Model Regarding Interpersonal Harmony Conflict

**FIGURE 1:**
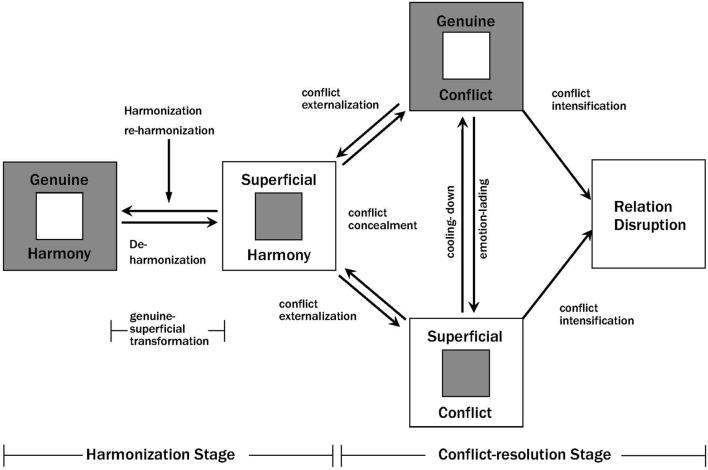
**A dynamic model of interpersonal harmony and conflict (disharmony) (Huang, 1999, p. 136)**.

Huang (1999) developed a formal psychological theory, the dynamic model of interpersonal harmony and conflict (**Figure 1**), based on real-virtual dialectics (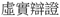) of the Yin–Yang perspective. In this model, interpersonal harmony and conflict are divided into *genuine/superficial* harmony and authentic/virtual focus conflict, whereas implicit/hidden conflict is regarded as superficial harmony. According to this framework, the state of harmony versus conflict between two people is dynamic and evolves within the web of their relationship.

*Genuine harmony* (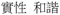) is defined as a relationship in which two parties concurrently sense tangible coordination, cooperation, congeniality, or even integration. By contrast, in *superficial harmony* (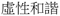; hidden disharmony, 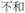), both parties attempt to maintain a deceptive outward harmony as a camouflage, but inwardly sense disharmony. Harmony between two parties can transform from genuine into superficial harmony as a result of changes in the similarity and differences between the parties (**Figure 1**).

Conflict, like harmony, can also be distinguished into *genuine* (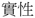) and *superficial* (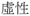) types, and this depends on whether the two parties involved focus on the core characteristics of the specific dispute or extend their conflict to encompass peripheral matters while concurrently allowing the negative emotions diffused during the conflict process to predominate. In a genuine conflict, the focus is on the specific matters underlying the existing differences of opinion and incompatible goals or concerns, and negative emotions are only a peripheral part of the conflict. By contrast, in a superficial conflict, the focus slowly drifts away from the specific problem or is extended, and negative emotions escalate gradually. Huang (1999) noted that a genuine conflict can transform into a superficial conflict through an **e*motionalizing process*** (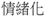; defocusing) and a superficial conflict can transform into a genuine conflict through a ***cooling-down process*** (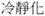; refocusing). Typically, genuine conflicts can be resolved more readily than superficial conflicts because of their relatively clearer focus on solvable problems, and thus the probability of the postconflict relationship between two parties being harmonious is also comparatively higher in the case of genuine conflict. Conversely, superficial conflicts are commonly over emotionalized and therefore more challenging to resolve as compared with genuine conflicts, and, correspondingly, the postconflict relationship in has a relatively higher probability of being only a superficial harmony or of completely breaking down.

Subsequently, Huang (1999) conducted a qualitative study involving in-depth interviews with 30 adults; in the study, Huang adopted the grounded-theory approach, used the previously developed dynamic model of interpersonal harmony and conflict as the formal model, and combined it with the Chinese concepts on the three aspects of harmony. Next, two essential theories regarding interpersonal harmony and conflict were constructed. In the first substantial theory, the meaning of various types of harmony and the relationships between the harmonies were developed. The results showed that in the case of *genuine harmony*, the parties involved in the relationship tend to hold positive perceptions of each other and typically interact in a sincere, trustful, active, supportive, accepting, and natural manner. Genuine harmony can be further distinguished into three subtypes, the rapport, affiliation, and role-fitting types. By comparison, in the case of *superficial harmony*, people tend to hold a more negative view of their relationship partners, and their behavior is commonly cautious, defensive, and ceremonious; accordingly, the reactions tend to be passive, rejecting, delaying, ignoring, and segregating. Moreover, superficial harmony, like genuine harmony, can be distinguished into three subtypes, the segmentation, alienation, and inhibition types. The *inhibition-type superficial harmony* (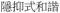) is the strongest form of unobservable conflict and can transform into explicit conflict at any time (**Table 1**; arrows indicate the direction of transformation).

**Table 1:** Various type of interpersonal harmony: Main characteristics and transformative relationship.

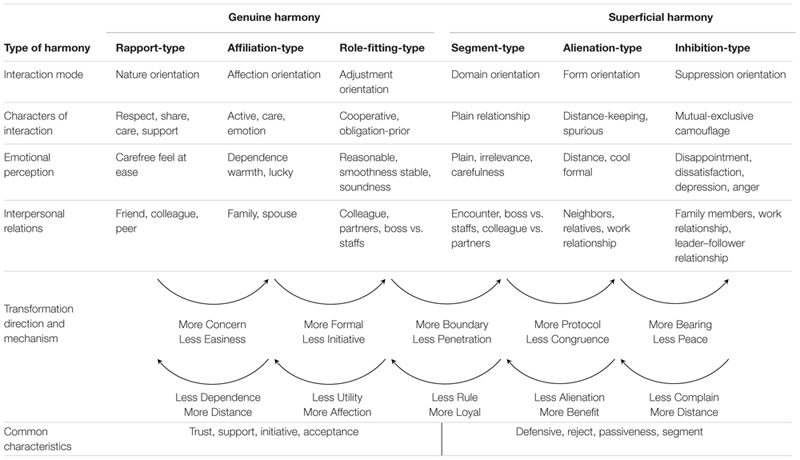

In the second substantial theory, interpersonal conflict was defined as a verbal or other visible behavior that openly expresses a difference of opinion, incompatible goals, requirements, or concerns, and the accompanying emotions; conflict was also classified into genuine and superficial types according to the level of focus on the issue and the degree of diffusion of emotion. *Genuine conflict* refers to a conflict in which the dispute is focused and emotion is diffused. When emotion is not diffused, a genuine conflict could transform into a superficial conflict, which would lead to a reduction in the clarity of the conflict focus. Here, *conflict* refers to the explicit type of conflict, and *implicit conflict* is equivalent to *superficial harmony.*

Huang (1999) further classified interpersonal conflict into three types of genuine conflict and three types of superficial conflict (**Table 2**; arrow: direction of transformation). A *Reasoning-Persuasion genuine conflict* (Type 1, 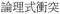), the most rational type of conflict, occurs when people engage in a debate with others to defend their values, or when they persuade others to agree to support their viewpoint. If the process becomes emotionally charged and loses focus on the contended point, the conflict might transform into a *Quarreling superficial conflict* (Type 4, 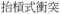); this type of conflict occurs when the dyad disputes orally with a high level of wit, repartee, tension, and competition, and the participants are unable to maintain emotional control and lose the point of the discussion. A *Contending genuine conflic*t (Type 2, 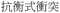) arises when people sense that they are being treated unfairly by others and thus fight for their own benefits or rights. If this type of conflict is over emotionalized, it might transform into a *Fighting superficial conflict* (Type 5, 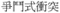); this conflict results when in order to win, each party uses all available resources, such as power, legal action, physical force, or even a weapon, with anger, rejection, rage, and hostility until the dispute is settled. A *Friction genuine conflict* (Type 3, 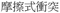) occurs either when people sense that their autonomy has been constrained or their intentions have been misperceived, or when one party is dissatisfied with the other. If the accumulated dissatisfaction from the past emerges during a current conflict, this type of conflict might transform into an *Entanglement superficial conflict* (Type 6, 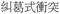), the most emotionally escalated type of conflict. The dispute leaves the problem unresolved and the participants filled with complaints, hate, disappointment, and a sense of helplessness.

**Table 2:** Genuine/superficial conflicts and their relationships.

Genuine Conflict (focus on the issue)	Transformation	Superficial Conflict (lack of focus, negative emotion)
T1.Reasoning/Persuasion	↔	T4. Quarreling type
↕		↕
T2. Contending type	↔	T5. Fighting type
↕		↕
T3. Friction type	↔	T6. Entanglement type

The dynamic model of Huang (1999) proposes that the degree of focus on a problem moves gradually from high to low from Type 1 to Type 6, whereas the degree of emotionality increases gradually from Type 1 to Type 6. Thus, conflict is at the lowest level in Type 1 and at the most escalated level in Type 6. The relationship between the two elements (problem and emotion) is identical to the relationship and transformation between Yin and Yang.

### Validating the Essential Theories of Interpersonal Harmony/Conflict

While the two essential theories were constructed from the qualitative study conducted using the grounded-theory approach, certain new concepts and their relationships were also developed. These essential theories also correspond to the discourses on the “relationalism” of Hwang (2000), which implies that distinct relationships entail divergent details of interpersonal harmony/conflict. Subsequently, the author conducted a series of quantitative studies to verify the construct validity or predictive validity of the two essential theories of dynamic model of interpersonal harmony and conflict.

#### Two Questionnaires for Testing the Construct Validity of Various Types of Harmony

According to the first substantial theory, the six basic types of interpersonal harmony and their main interaction rules were systematically investigated (**Table 2**). In *Natural-oriented* (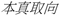) interaction, both parties in a relationship present their natural “persona” and show mutual acceptance and respect in all scenarios, and this interaction frequently occurs in rapport harmony (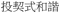). In *Affection-oriented* (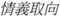) interaction, one party assigns higher priority to the other party’s requirements than to its own, even if this costs the first party in terms of personal suffering, and this interaction invariably occurs in affiliation harmony (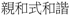). *Adjustment-oriented* (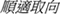) interaction mostly appears in role-fitting harmony (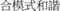), and in this interaction, the two parties follow their own roles carefully, although affection will occasionally be involved in making the relationship between the parties smooth. In *Domain-oriented* (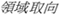) interaction, the parties aim to simplify their relationship and avoid becoming involved with irrelevant events; this interaction mostly appears in segmentation harmony (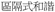). In *Form-oriented* (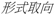) interaction, the parties interact with each other in a plain manner and maintain a light and almost spurious relationship; this interaction mostly appears in alienation harmony (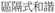). Lastly, inhibition harmony (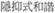) invariably leads to *Suppression-oriented* (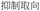) interaction, in which one party must suppress its anger or dissatisfaction toward the other party, because failure to do so will offend or cause an argument with the other party.

To verify the construct validity of the six types of harmony, two self-report questionnaires were designed and each was administered to approximately 250 adults (total 500) living in northern Taiwan (Huang, 1999). Six scenarios were presented to demonstrate each of the six types of harmony, and six statements were also prepared to indicate the aforementioned six types of oriented interactions related to distinct types of harmony. In the questionnaire, each scenario was presented first, and this was followed by a series of questions on the scenarios and the oriented interactions. Moreover, background information on the participants was collected. Two versions of the questionnaire were used, each questionnaire included three scenarios each of genuine harmony or superficial harmony, and the order effect was balanced in the questionnaires.

The result obtained in the study supported the construct validity of the six types of interpersonal harmony (**Table 3**). The statistical values along the diagonal in **Table 3** are higher than the adjacent statistics, which supports the conclusion that the main harmony was correlated more closely with the main oriented interaction and was less correlated (irrelative) with other oriented interactions. The results also indicated that various types of harmony exist according to distinct relationships and concerns, and further that maintaining superficial harmony is more common than conflict externalization among Chinese people.

**Table 3:** Interaction orientation under six types of interpersonal harmony.

Interaction-oriented	Genuine Harmony (*n* = 248)			Superficial Harmony (*n* = 252)		
	Rapport-type (*n* = 214)	Affiliation-type (*n* = 205)	Role-fitting type (*n* = 170)	Segment-type (*n* = 231)	Alienation-type (*n* = 190)	Inhibition-type (*n* = 81)
Nature-oriented	**174^a^ (81.3)^b^**	74 (36.1)	48 (28.2)	29 (12.6)	13 (6.8)	8 (9.9)
Affection-oriented	20 (9.3)	**91 (44.4)**	28 (16.5)	7 (3.0)	7 (3.7)	11 (13.6)
Adjustment-oriented	17 (7.9)	26 (12.7)	**76 (44.7)**	69 (29.9)	45 (23.7)	16 (19.8)
Domain-oriented	2 (0.9)	6 (2.9)	7 (4.1)	**80 (34.6)**	48 (25.3)	14 (17.3)
Form-oriented	1 (0.5)	2 (1.0)	5 (2.9)	36 (15.6)	**63 (33.2)**	15 (18.5)
Suppression-oriented	0	6 (2.9)	6 (3.5)	10 (4.3)	14 (7.4)	**17 (21.0)**

*a, number of sample; b, percentage.*

#### Laboratory Test for Validating the Various Types of Interpersonal Conflict

Huang’s model is a dynamic model (rather than a static or structural model) focused on explicit but not implicit conflict, and methods involving the use of a structured and self-reported questionnaire cannot demonstrate the features of dynamic conflict. Consequently, a **laboratory** study imitating the strange situation experiment, which was originally developed to classify attachment relationships in children (Ainsworth et al., 1978), was conducted to examine the validity of various types of interpersonal conflict (**Table 2**).

The study was designed to observe the interaction process during the conflict between a parent and a child, as well as the parent’s parenting style (Huang and Huang, 2002). Most parent–child conflicts in Chinese society occur during the parents’ attempts to guide or monitor their children’s homework and daily habits. Parents expect their children to cooperate and follow rules, whereas children seek to either engage in activities that they enjoy, such as watching TV or playing video games, or simply behave as they wish to. In a typical Chinese family, the mother is most frequently responsible for monitoring the child’s schoolwork.

In the study, the aim was to design a paradigm to revalidate Huang’s dynamic model of conflict in a mother-and-child interaction based on the following three assumptions. First, although the six basic types of conflict were derived from adult samples, the six types of conflict should also be identifiable in the relationship between mothers and young children. Second, if conflicts occur in a seminatural mother–child supervisory scenario, as pressure gradually increases, conflicts should typically develop with increased ease and become progressively more emotionalized. Third, if various types of mother–child conflict can be successfully classified, the emergence of four prototypes of mother–child conflict (combinations of genuine/superficial and mother/child) should be identifiable.

The participants were 45 pairs of mothers and sons or daughters living in southern Taiwan. All children were fourth-grade students in elementary school, and the study included an almost equal number of boys and girls. The mothers’ ages ranged from 30 to 49 years old and averaged 39 years and 8 months. The average length of marriage was 13.6 years. Most of the mothers enrolled in the study were the primary caregivers of the children.

A semi-natural scenario was designed to induce mother–child conflict. Based on previous research and observations, in the scenario established, the mothers could monitor their children doing homework in our laboratory, and pressure was gradually increased to induce conflict. Observation of the mothers’ handling of the conflict was the key to understanding the mothers’ attitudes toward parenting.

Each mother–child pair was required to complete three tasks of distinct levels of complexity: sorting materials (low complexity), solving a puzzle (medium complexity), and completing mathematical exercises (high complexity). The problem in each task was solved by the mother and the child working together or by the child working alone under the mother’s direction and supervision. The procedure was divided into six stages, with the stress level being increased gradually across the stages to provoke conflict. To induce conflict between mother and child, distraction elements and time pressure were included. The distractions were cartoon videos for the child and the filling out of a family information questionnaire for the mother. When the child was distracted from the task, the mother was required to urge the child to concentrate. Conversely, when the mother had to both work on her task and supervise the child’s work, she was placed under added pressure and thus more readily provoked into conflict than when not under pressure. Time pressure was applied in the form of informing participants of the amount of working time remaining, and this would result in the mother urging the child to complete the task. **Table 4** displays the design of the mother–child conflict procedure used in the laboratory.

**Table 4:** Mother–child conflict situations and procedures.

Stage	Task	Cooperation or Supervision	Benchmarking	Distraction	Duration (minutes)
1	Low complexity task: sorting materials	Mother–child cooperation	None	None	3
2		Mother directs child in task	None	Child: cartoon	3

3	Medium complexity task: puzzle	Mother–child cooperation	None	Child: cartoon	5
4		Mother directs child in task	Time pressure	Child: cartoon	5

5	High complexity task: mathematical exercises for grade 2 or 3	Mother–child cooperation	Time pressure	Child: cartoon	7
6		Mother directs child in task	Time pressure	Child: cartoon Mother: questionnaire	7

Total					30

A workbook for the experiment was organized and compiled after the pretest; the workbook contained detailed information on the experimental procedures, methods, and working definitions used for observation and classification of mother–child conflict. The formal test involving the 45 pairs of participants was then conducted according to the workbook. Two observers monitored the behavior of the mother and the child separately from behind a one-way mirror through all stages of the trials, and all trials were video-recorded. Each observer coded the behaviors of the participants and, after each trial, classified the type of mother–child conflict.

Mother–child conflict was classified based on the dynamic model of interpersonal harmony and conflict of Huang (1999); as noted in preceding sections, the classification system is based on transformations derived from Yin–Yang dialectics (**Table 2**). The details of the operational definition of classification and the experimental procedure have been described previously (Huang and Huang, 2002).

The results obtained were the following: almost half of the study participants (44.4% of the children and 46.7% of the mothers) started to engage in conflicts during the first two stages of the trial; more than half (51.1%) of the participants reached the peak of conflict at the fifth stage; and the small increments of pressure applied according to the experimental procedure were adequate for intensifying the mother–child conflict. The results also revealed that with an increase in the applied pressure, genuine conflict transformed into superficial conflict and reached its peak during the last two stages of the trial, and further that the conflict became progressively less focused and more emotionalized.

Other results of the study showed that in the case of mothers, 80% of the initial conflicts were of the reasoning-persuasion type, whereas for children, 62.2 and 35.6% of the initial conflicts were of the contending and reasoning-persuasion types, respectively. As anticipated, mothers maintained focus on genuine conflict to a greater extent than the children did at the initial stage of the trial. Conflict was commonly triggered by a mother’s insistence that her opinion or judgment be followed by the child (persuasion-type conflict). When persuading a child to agree with her opinion or follow her directions, the mother initiated verbal conflict with the child, and at this point, there was comparatively less emotional argument and more guidance. However, the children frequently assumed that they were being treated unfairly, or that their wellbeing was not being considered (contending-type conflict). Lastly, 86% of the mothers maintained genuine conflict (i.e., most did not become angry), but in the case of the children, 22.2% of the conflicts evolved into superficial types of conflict, among which fighting conflict appeared most commonly. Thus, the children lost control of their temper more frequently than the mothers did.

The study demonstrated that all six types of conflict predicted by the model could be successfully induced using the quasi-experimental design, and further showed that genuine versus superficial conflict can be reliably observed and classified, much like the transformation between the distinct types of conflict. The study also indicated that a small amount of experimentally induced pressure is adequate for provoking dyadic conflict, and that as the pressure is increased, such a conflict can transform from an issue-focused conflict into a conflict in which focus is lost and emotions escalate. These results validated the dynamic model of interpersonal harmony and conflict. This study represents the first attempt to experimentally operationalize a dynamic model of conflict that originated in indigenous Chinese theory. Because the mother–child relationship can be considered the most elementary of all relationships, the prototypes identified here could be applied validly and effectively in diverse dyadic conflicts involving other types of actors and other types of relationships.

The aforementioned study also presents key implications for indigenous Chinese psychology. The Yin–Yang dialectic is a unique and central element of traditional Chinese thought; however, because of the complexity of the transformations between Yin and Yang, previous researchers have found it extremely challenging to operationalize them into a quantifiable psychological theory. The research discussed in this section represents a first step in showing that a science of transformations can potentially be developed. Such a science would hold critical implications for conflict resolution. When conflict and harmony are conceptualized as ongoing sequences of dialectical transformations between problem focus and emotionalization, it represents the identification of the current stage of the process and its prototypical sequences of change that are most relevant for conflict resolution, and not the determination of the existence or absence of a particular cause and effect. Traditional Chinese thought was centered on the concept of transformations occurring over time, not on the isolation of cause and effect. The type of contribution that such an indigenous approach can make to social science remains to be ascertained.

## Extension of “Dynamic Model of Interpersonal Harmony and Conflict”

After verifying the construct validity of the concepts of harmony and conflict in the dynamic model of interpersonal harmony and conflict, studies were conducted to both verify the dynamic model and concurrently apply the model to various relationships and distinct cases of harmony and conflict.

### Subordinate–Supervisor Conflict in the Chinese Workplace

A literature review reveals that the bidimensional five-style framework of conflict resolution (avoiding, accommodating, collaborating, competing, and compromising) is now universally accepted (Thomas, 1976; Rahim, 1986). Moreover, even the so-called win–win and lose–lose types of concepts have been derived from this structure, which has also been confirmed by several cross-cultural studies. All previous cross-cultural studies have supported the concept that in a collectivist culture, people tend to adopt the compromising, accommodating, obliging, or avoiding styles of conflict resolution, whereas in individualist cultures, people commonly adopt direct, competitive, and dominating styles of conflict resolution (Ting-Toomey et al., 1991; Trubisky et al., 1991; Weldon and Jehn, 1995).

However, recent research suggests that people typically do not use only a single conflict-resolution style when handling interpersonal conflict: they instead adopt a combination of several styles (Munduate et al., 1999), termed *conglomerated conflict behavior* or *patterns of conflict resolution*, which has not been tested in cross-cultural studies.

Western studies have indicated that the relationship between two parties involved in a conflict plays no role in the pattern of conflict resolution (Munduate et al., 1999). By contrast, a theory developed in a culturally Chinese context commonly treats relationships as a major factor when determining conflict-resolution patterns. In addition to emphasizing the dynamic nature of conflict, the dynamic model of harmony and conflict stresses the role of relational context in the conflict-resolution style of culturally Chinese people (Huang, 1999; Leung et al., 2002).

The study conducted by Huang et al. (2007) integrated the dynamic model of harmony and conflict of Huang (1999) with the commonly accepted five-style framework of conflict resolution in order to analyze and test the process of multistyle dynamic conflict resolution. In the study, the first requirement was to confirm that the patterned conflict-resolution behaviors could be observed and to identify the specific patterns of conflict-resolution behavior in a culturally Chinese society. The second requirement was to determine whether the type of relationship between two parties in a conflict influences the selection of the conflict-resolution style, and whether the patterns of conflict resolution affect the subsequent relationship.

In the study, the critical incident technique (Flanagan, 1954) was used to collect instances of common conflict between supervisors and subordinates. This technique is well-suited for examining conflict as a dynamic process because it can capture conflict issues and scenarios, the reactions of each party, and the results of the conflict-resolution style(s) applied.

Culturally Chinese people commonly hold a negative attitude toward conflict (Huang, 1999), and thus they might not wish to describe conflicts with their supervisors to strangers. Even where a large sample size is obtained through the use of questionnaires, the study participants might not include adequate details regarding such conflicts. To avoid this drawback, all data for this study were collected through personal interviews conducted in a private setting. The interviewees were 23 people in Taiwan (13 males), aged between 25 and 47 years old, who reported a total of 28 instances of conflict, 23 of which were conflicts with supervisors. Each participant reported at least one conflict with a supervisor.

The results showed that in 78% (18/23) of the reported instances, people adopted more than one conflict-resolution style to resolve the dispute. This result is similar to the results from Western studies. Thus, the concept of conglomerated conflict resolution also applies in the case of culturally Chinese people. Two main patterns of conflict behavior were affirmed: “direct encounter followed by compromise or coordination” and “direct encounter followed by avoidance.”

The most prevalent pattern of conflict resolution identified was direct encounter followed by compromise. The people who adopt this pattern first seek to achieve their own target or have their demands met or opinions accepted, and failing that, they attempt to reach an agreement in order to create a win–win scenario. Both the avoiding and accommodating strategies require forgoing one’s own target or demand. These two strategies could be combined to create the second major pattern, direct encounter followed by avoidance. In this pattern, subordinates initially present their own demands or opinions but eventually discard them, which creates a win–lose scenario. This pattern has not been identified in Western studies (Munduate et al., 1999).

In more than two-thirds of the cases, the prevailing reaction to conflict with a supervisor was neither accommodation nor avoidance. This result clearly differs from the previous finding that culturally Chinese people tend to adopt avoiding or accommodating conflict-resolution styles (Trubisky et al., 1991). The result is also distinct from the finding reported by Hwang (1997–1998) that obliging is the dominant reaction in a vertical conflict, particularly in the case of a child–parent or subordinate–supervisor conflict. However, the results of this study (Huang et al., 2007) agree with those of a study conducted by Huang et al. (2005), which determined that Taiwanese employees typically use collaboration or compromise to handle workplace conflicts.

When analyzing the study data, new insights were gained through an examination of how preconflict and postconflict relationships between supervisors and subordinates were related to the conflict-resolution patterns adopted. The results showed that when the superior–subordinate relationship was a genuine harmony, the direct-encounter or the direct-encounter-then-compromise pattern was highly likely to be adopted; this means that subordinates expressed their own opinions and attempted to reach a compromise or used integration as the solution to problems. When the subordinates stated their own opinion, although the conflict was typically resolved through accommodation or avoidance, the postconflict relationship typically continued to be of the genuine harmony type. By contrast, when the preconflict relationship was a superficial harmony, subordinates were comparatively less inclined to expose the conflict, and they commonly adopted either the accommodating or avoiding style or the direct-encounter-then-yield pattern to handle the conflict. The postconflict relationship here fell under the inhibition subtype of superficial harmony. Thus, the nature of the preconflict relationship affects the style and pattern of conflict resolution, and then continuously influences the postconflict relationship.

Conversely, if the preconflict relationship is considered to affect the type of conflict, then the type of conflict could be expected to influence the postconflict relationship. To further analyze the study data, explicit conflict was classified into the following six types based on the dynamic model for harmony and conflict proposed by Huang (1999): three types of genuine conflict (reasoning, contending, and friction), which are clearly issue-focused; and three types of superficial conflict (quarreling, fighting, and entanglement), which are highly emotional and not issue-focused.

The analysis results showed that half of the reported conflicts were genuine conflicts and were triggered by matters of reasoning, power or privilege, or resource allocation. These conflicts tended to remain rational and focused when the preconflict relationships were of the genuine harmony type, and then the postconflict relationship also continued to be a genuine harmony. Similarly, when preconflict relationships were of the superficial harmony type, the postconflict relationship continued to be a superficial harmony. However, in some of these cases in which the preconflict relationships were of the superficial harmony type, the parties became emotional and the conflict lost issue-focus and transformed into a superficial conflict. The fighting type of conflict was the main type of conflict that developed. In cases in which the conflict became superficial (fighting or quarreling type), the postconflict relationships remained at the level of superficial harmony. Thus, the preconflict relationship appeared to exert a stronger effect on the postconflict relationship than the conflict type did. Four instances of hidden conflict (inhibition harmony) with the supervisor were also reported in the study. The participants involved might have strongly disagreed with their supervisors on certain matters but not have exposed this, and thus the supervisors might not have been aware of the disagreement. Subordinates in this type of a scenario typically harbor strong negative feelings toward, and keep their distance from, the supervisors. Consequently, morale was low among these four participants.

The “direct encounter followed by compromise or coordination” pattern of conflict resolution allowed genuine harmony to flourish in the postconflict relationship. Conversely, the “direct encounter followed by avoidance” pattern resulted in superficial harmony in the postconflict relationship. Lastly, the accommodating style tended to cause the conflict to remain implicit, and thus only the inhibition type of superficial harmony remained in the relationship.

In summary, this study demonstrated that if an emic approach is adopted, particularly using the Huang’s model on interpersonal harmony and conflict, to investigate conflict-resolution patterns in the workplace, numerous insights can be gained that are considerably distinct from those yielded by the results obtained using an etic approach, and that this should provide a new conflict-management strategy for Chinese organizations.

### Hidden Conflict and Harmonization Mechanism in the Relationship between Mothers- and Daughters-in-Law: From Superficiality to Genuineness

The most complex vertical relationship in Chinese society is the one between mothers- and daughters-in-law, which has long been described as “the war between two women.” Studies to date on the relationship between mothers- and daughters-in-law have emphasized the static rather than the dynamic conflicts between them. However, one previous study (Huang and Hsu, 2006), which is discussed here, used the dynamic model of interpersonal harmony and conflicts of Huang (1999), a model that is highly favorable for understanding this relationship and can precisely portray the progress in the transformation of the relationship.

Analysis of the data gathered from 19 in-depth interviews revealed an extensive range in the harmony between mothers- and daughters-in-law. The findings revealed that the relationship between a mother-in-law and her daughter-in-law is initially a superficial harmony, because they interact with each other through the same person but do not interact directly. The harmonization process begins with both parties avoiding face-to-face conflicts and maintaining superficial harmony during the beginning stages of the marriage, following which the relationship changes depending on whether the mother- and daughter-in-law change their ***obliffection*** (***chin-yi*,
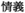
**), which refers to obligations and affections involved in the interaction leading toward the relationship.

If the mother- and daughter-in-law wish to maintain a relationship of genuine harmony, their outward behaviors must closely match their hidden emotions. Outer behaviors within this genuine harmony invariably transform the hidden emotions into progressively more positive emotions. Positive hidden emotions and outer behaviors make this form of harmony a dynamic progression that includes three phases in terms of the distinct intensities of the relationship quality and the harmonization mechanism of dynamic interaction in the transformation process. In Phase 1 (formation of genuine harmony), a relationship of the genuine harmony type begins to develop as each person fulfills her mutual obligations as a mother- or daughter-in-law. In Phase 2 (maintenance of genuine harmony), the mothers- and daughters-in-law freely express affection for one another and identify transforming solutions to hidden conflict, and their genuine harmony relationship at this stage is steady. In Phase 3 (refinement of genuine harmony), the genuine harmony is reinforced as the relationship becomes progressively more interactive, and the parties no longer focus on merely fulfilling their obligations as mothers- and daughters-in-law. Conversely, if the relationship continues to remain a superficial harmony, the interactions and their consequences differ completely from those in a genuine harmony.

The study revealed that obliffection (*chin-yi*) is the core harmonization mechanism operating in the relationship between mothers- and daughters-in-law. Therefore, Hsu and Huang (2006) confirmed that the traditional optimal relationship between mothers- and daughters-in-law is the affiliation type of harmony, which indicates that the core interaction mode is the affective-orientation mode and that it also involves active caring emotion. However, in the case of present-day mothers- and daughters-in-law, the optimal relationship they seek might be a role-fitting-type of harmony, which suggests that adjustment-orientation, cooperation, and obligation priority are highly favorable (Hsu and Huang, 2006).

### Hidden Conflict or Ignoring in Adolescent Friendships

Hidden conflict is more widespread than explicit conflict in Chinese society, as suggested by the theory of Huang (1999). Previous Western studies have treated ignoring (hidden conflict) as one type of relationship aggression. However, ignoring (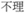) and ending of relationships occur in close friendships. From the relationship aggression viewpoint, no explanation is available for why the actor senses pain and guilt in this scenario. Chinese people emphasize relationships, and thus they cannot readily confront conflict and prefer to allow a conflict to become implicit. Instead of damaging a relationship explicitly and directly, ignoring the target friend is one approach used for coping with interpersonal conflict and leading the relationship into a superficial harmony: Ignoring silently conveys a sense of dissatisfaction and reduces intimacy in a friendship. Therefore, Lai and Huang (2013) examined the meaning and process of ignoring (hidden conflict) in adolescent friendships in a previous study by adopting the dynamic model of interpersonal harmony and conflict, which is based on the Chinese cultural context rather than on the relational aggression viewpoint.

In the study (Lai and Huang, 2013), data on past instances of ignoring experiences were gathered by interviewing 14 participants (13–29 years old; 11 females). The results showed that the ignoring process was a practice of the method of balancing the “I-Thou” psychological distance. Close friendships in adolescence are high-support relationships but can be unstable. The role obligations between friends are obscure, and the causes of conflict in these relationships are also illegitimate, such as “unwittingly harm,” “inequitable affections,” “anger transferring,” and “more independent space.” Conversely, the cultural demand for maintaining harmony results in a dilemma in which by acting out, a person harms the other person in the relationship, but by not acting out, one harms oneself. When faced with this “stuck in the mud” or “lose–lose scenario” period, four paths were followed by the study participants.

If the conflict remained vague, the relationship entered into a superficial harmony in which the intimacy between the parties decreased. If the conflict escalated, the relationship became broken. If an opportunity presented itself for communicating clearly and expressing the value of each partner in the relationship, the relationship developed into a genuine harmony.

Lastly, in certain cases, even after the contact between friends ceased to exist, the people involved did not readily accept or become aware of the end of the relationship, which suggests that the consequences of ignoring were not all negative. However, from the relationship aggression viewpoint, no positive results can be observed here.

### Emotion Sharing and Its Effect on Superficial Friendships Developing into Genuine Friendships

Previous research on friendship mostly subscribed to the “social penetration theory” and considered that the interacting dyads progressively disclosed themselves through daily interaction and developed intimacy gradually. The theory of Huang (1999) suggests that attention must be devoted to the superficially harmonious aspect of friendship. Friends in a superficial harmony might conceal their disagreements, and even when they claim the other as “a friend,” they might not genuinely care for each other, and they will also not be willing to engage in a close mutual friendship voluntarily. According to the self-disclosure theory, a superficial friendship appears to remain frozen in this state forever. However, this view has been called into question.

A series of studies attempted to examine whether the automatic process of “social sharing of emotion” (Rime et al., 1991) might result in a change or breakthrough that leads to a superficial-harmony friendship developing into a genuine-harmony friendship (Huang and Huang, 2012).

In Study 1, questionnaires were answered by 383 Taiwanese college students. The results showed that emotion sharing occurred in both superficial- and genuine-harmony friendships in Chinese society and that distinct types of harmony displayed the same sharing pattern. All types of emotion were shared, and the friendship quality was affected after the sharing of emotions. Study 2 was focused on the types of emotion shared in a superficial harmony that appeared at a low frequency in Study 1, and the in-depth interview was used as the qualitative study method. The outcome revealed that emotion sharing in a superficial friendship can potentially end the superficial relationship itself, and that the main influencing factors could be “change in impression,” “the same feeling,” and “feeling of secret sharing.” To further examine the findings from these two studies, a quasi-experimental method was used in Study 3. The experimental stories included four manipulated emotions, sadness, happiness, guilt, and affection, plus one control condition, non-emotion sharing. The results indicated that friendship quality was affected by emotion sharing and that the change was moderated by the previous harmony type. Furthermore, in a superficial friendship, the effect of negative-emotion sharing on friendship quality was mediated by “feeling of secret sharing,” which did not occur in the case of positive-emotion sharing.

The findings of this series of studies clearly reveal that the emotion sharing theory is more appropriate than the self-disclosure theory for interpreting the breakthrough that results in a superficial-harmony friendship developing into a genuine-harmony friendship.

## Conclusion and Discussion

Most Western studies on conflict are based on the social exchange theory, conflict dialectics, and PDG. Because Chinese culture or political ideology emphasizes the value of harmony and devaluates conflict, a harmony theory is specially required for understanding Chinese people (i.e., an indigenous Chinese theory that considers harmony and conflict concurrently is necessary).

Based on the Yin–Yang perspective or the dialectic of harmonization, the author first reconstructed and reinterpreted the concepts regarding harmony and conflict embodied in traditional Chinese thought, specifically the theory of integration of heaven and humanity, the theory of rituals (*Li*-thoughts), and the state-ideological Confucianism, which imply, respectively, the ideas of individual personality, interpersonal ethics, and the interests and effectiveness of groups or organizations. Concomitantly, the author established a formal psychological theory/model, “the dynamic model of interpersonal harmony and conflict,” based on the Yin–Yang perspective/dialectic of harmonization/real-virtual dialectics, which reflected the basic views of Confucianism and Taoism. This model referred to genuine versus superficial harmony and authentic versus virtual focus conflict, but it regarded implicit/hidden conflict as superficial harmony. Based on this formal model, a qualitative study was conducted to construct, respectively, substantial theory regarding interpersonal harmony and conflict. Lastly, to validate this model, the author conducted a series of quantitative and extensive studies on interpersonal harmony/conflict within the relationships between parents and children, supervisors and subordinates, mothers- and daughters-in-law, and friends, and the results provided new insights into various types of relationships.

In summary, the work discussed in this article demonstrates that a formal psychological theory/model can be developed based on the Yin–Yang perspective. Furthermore, the described findings have revealed that by combining qualitative and quantitative empirical research, a substantial theory can be established and the concepts can be validated. Thus, a Chinese indigenous psychology rooted in traditional cultural thought and Eastern philosophy has been realized.

Additionally, this article might raise certain arguments in the Chinese indigenous research field. First, the meaning of Yin–Yang is highly controversial. Yin–Yang, as an indigenous Chinese notion, could be regarded as a type of Chinese philosophy (Feng, 1983). “Philosophy” means the love of wisdom, and the modern science of philosophy originated in the West. Yin–Yang, as a traditional Chinese philosophy, lacks methodology and operationalizable methods. However, Li X. (2014) argued that Yin–Yang is one type of logic system (the other three being Aristotle’s formal logic, Bohr’s complementarity logic, and Hegel’s dialectic logic) and could be applicable and powerful in certain contexts. Thus, Li X. (2014) suggested that Yin–Yang might inspire but cannot guide Chinese indigenous research. By contrast, Li (2012) proposed that Yin–Yang must be viewed as one of three basic cognitive frames (the other two being Aristotle’s formal logic and Hegel’s dialectical logic). Furthermore, Li P.P. (2014) noted that Yin–Yang as an epistemology contains three core tenets, “holistic content,” “dynamic process,” and “duality integration,” as well as three operating mechanisms, “asymmetrical balancing,” “transitional balancing,” and “curvilinear balancing.” Therefore, Li P.P. (2014) suggested that “Yin–Yang Balance” is of unique value for Chinese indigenous management research and its application to the case-study method in particular (Li P.P., 2014).

The author agrees with Ping Li’s viewpoint and regards Yin–Yang as an epistemology and as being context-specific. Because Yin–Yang is an epistemology, the author used the Yin–Yang perspective to develop the formal model. Moreover, because Yin–Yang is context-specific, the author used the Yin–Yang perspective under the context of harmony and conflict, and then adopted the viewpoint of Cheng (1977), according to which the dialectic of harmonization is regarded as one of metaphysics (the other two being Hegel’s dialectics of conflict and Madhyamika dialectics). As a metaphysics that includes ontology, cosmology, and natural philosophy and denotes a non-empirical type of philosophical enquiry into the nature of existence, the dialectic of harmonization can serve as a new perspective on culture (Huang, 2008; Fang, 2012).

Thus, considering Yin–Yang as an epistemology might not only inspire research on traditional thought/philosophy but also integrate the philosophy into modern scientific research. Although Yin–Yang was regarded as an epistemology and the formal model of interpersonal harmony and conflict was constructed based on this epistemology, the model corresponds to the three core tenets of the Yin–Yang frame proposed by Li P.P. (2014). Moreover, the research extended previous work and confirmed two of the three operating mechanisms of Yin–Yang balancing, asymmetrical balancing and transitional balancing, and only the mechanism of curvilinear balancing remains to be confirmed.

The Huang’s (1999) dynamic model of interpersonal harmony and conflict also inspired certain advanced concepts and research in the conflict management field. Leung (1988) initially reported that conflict avoidance is more common in the East Asian society than in Western society; however, in contrast to this, Leung subsequently constructed a dualistic model of harmony (harmony enhancement and disintegration avoidance) and combined conflict and thus suggested an integrated model for conflict management (Leung et al., 2002; Leung and Brew, 2009). Furthermore, the new concepts were used, without being regarded as indigenous concepts, for conducting a cross-cultural study (Leung et al., 2011).

The author’s dynamic model of interpersonal harmony and conflict rooted in Yin–Yang dialectics is a monocultural theory (Yang, 1999) or an “Eastern *emic-*as-*emic*” type of indigenous research (Li, 2012), and thus the model and the outcomes from related studies are highly suited to Chinese society. According to the notion of Li P.P. (2014), although the Yin–Yang frame was originated from Chinese culture, it can be applied to people from all cultures. Brew (2007) perceived a requirement to integrate Western and Chinese conceptual frameworks into a single model that would be ontologically meaningful for Western and Asian study samples; however, Brew still focused on conflict and not on harmony. In this respect, whether the Huang model can be applied to international societies must be investigated further in cross-cultural comparisons. Regardless of the type of current research being conducted on conflict management, an emerging trend has been the incorporation of the Chinese notion of harmony into conflict management, and the adoption of Yin–Yang as a new perspective on culture in the field of management.

## Author Contributions

The author confirms being the sole contributor of this work and approved it for publication.

## Conflict of Interest Statement

The author declares that the research was conducted in the absence of any commercial or financial relationships that could be construed as a potential conflict of interest.
